# The Hospital Sunday Fund

**Published:** 1913-06-28

**Authors:** 


### The Hospital Sunday Fund.

JUNE 20, 1913

The following are among the amounts received at the Mansion House to date :—

| | | | | | | |
|---|---|---|---|---|---|---|
| Christ Church, Lancaster Gate | | | | £839 | 0 | 0 |
| St. Mary Abbot's, Kensington | £188 | 14 | 6 | | | |
| *with* Christ Church, Victoria Road | 46 | 14 | 8 | | | |
| *and* St. Paul's Vicarage Gate | 76 | 7 | 1 | | | |
| Total, St. Mary Abbot's | | | | 311 | 16 | 3 |
| St. George's, Hanover Square (with St. Mary's, Bourdon Street) | | | | 264 | 0 | 0 |
| Essex Church, Kensington | | | | 236 | 0 | 0 |
| St. Stephen's, South Dulwich | | | | 167 | 0 | 0 |
| Westminster Abbey | | | | 119 | 0 | 0 |
| Brockwell Park Open-Air Mission | | | | 116 | 0 | 0 |
| Christ Church, Gipsy Hill | | | | 107 | 0 | 0 |
| Corporation of London (donation) | | | | 105 | 0 | 0 |
| St. Peter's, Brockley | | | | 101 | 0 | 0 |
| Brompton Parish Church | | | | 98 | 0 | 0 |
| Bromley (Kent) Parish Church with Chapel of Ease and Mission | | | | 96 | 0 | 0 |
| St. John's, Kensington | | | | 89 | 0 | 0 |
| Liberal Jewish Synagogue, St. Marylebone | | | | 83 | 0 | 0 |
| St. Andrew's, Leytonstone | | | | 80 | 0 | 0 |
| St. Luke's, West Hampstead | | | | 79 | 0 | 0 |
| All Saints', Ennismore Gardens | | | | 68 | 0 | 0 |
| St. Mary's, Kilburn | | | | 67 | 0 | 0 |
| St. Luke's Parish Church, Chelsea | | | | 62 | 0 | 0 |
| St. Augustine's, Kilburn | | | | 55 | 0 | 0 |
| St. Paul's, Beckenham | | | | 53 | 0 | 0 |
| St. John's, Forest Hill | | | | 51 | 0 | 0 |
| St. Saviour's, Ealing | | | | 51 | 0 | 0 |
| Bromley (Kent) Congregational Church | | | | 44 | 0 | 0 |
| Chipping Barnet Parish Church | | | | 37 | 0 | 0 |
| St. Andrew's, Westminster | | | | 32 | 0 | 0 |
| St. Paul's, Harringay | | | | 32 | 0 | 0 |
| St. George's, Campden Hill, W. | | | | 31 | 0 | 0 |
| All Saints', Clapham | | | | £26 | 0 | 0 |
| St. Agnes, Kennington Park | | | | 26 | 0 | 0 |
| St. John's, Kingston Vale | | | | 26 | 0 | 0 |
| Crayford Parish Church | | | | 25 | 0 | 0 |
| St. Luke's, Hornsey | | | | 24 | 0 | 0 |
| St. Mary's, Ewell | | | | 24 | 0 | 0 |
| St. Andrew's, Upper Norwood | | | | 23 | 0 | 0 |
| St. Olave's, Hart Street, E.C. | | | | 22 | 0 | 0 |
| St. Matthew's, Redhill | | | | 22 | 0 | 0 |
| St. Mary's, Plaistow | | | | 20 | 0 | 0 |
| St. Mary's, Cuddington | | | | 20 | 0 | 0 |
| St. Stephen's, Hampstead | | | | 20 | 0 | 0 |
| Total available for distribution to date, about | | | | 42,500 | 0 | 0 |

